# CD137-CD137 Ligand Interactions in Inflammation

**DOI:** 10.4110/in.2009.9.3.84

**Published:** 2009-06-30

**Authors:** Byungsuk Kwon

**Affiliations:** School of Biological Sciences, University of Ulsan, Ulsan, Korea.

**Keywords:** CD137, CD137 ligand, inflammation

## Abstract

The main stream of CD137 studies has been directed to the function of CD137 in CD8^+^ T-cell immunity, including its anti-tumor activity, and paradoxically the immunosuppressive activity of CD137, which proves to be of a great therapeutic potential for animal models of a variety of autoimmune and inflammatory diseases. Recent studies, however, add complexes to the biology of CD137. Accumulating is evidence supporting that there exists a bidirectional signal transduction pathway for the CD137 receptor and its ligand (CD137L). CD137/CD137L interactions are involved in the network of hematopoietic and nonhematopoietic cells in addition to the well characterized antigen-presenting cell-T cell interactions. Signaling through CD137L plays a critical role in the differentiation of myeloid cells and their cellular activities, suggesting that CD137L signals trigger and sustain inflammation. The overall consequence might be that the amplified inflammation by CD137L enhances the T-cell activity together with CD137 signals by upregulating costimulatory molecules, MHC molecules, cell adhesion molecules, cytokines, and chemokines. Solving this outstanding issue is urgent and will have an important clinical implication.

## INTRODUCTION

CD137 (also known as 4-1BB and TNFRSF9) is a prototype of costimulatory molecules for T cells. In particular, the interactions between CD137 and its ligand, CD137 ligand (CD137L; also known as 4-1BBL and TNFSF9), have been the most extensively studied in CD8^+^ T cells (1-3). Ligation of CD137 on CD8^+^ T cells has been shown to markedly increase their survival, proliferation, and cytotoxic T lymphocyte (CTL) activities (4,5). In support of the importance of these interactions in controlling CD8^+^ T-cell function, anti-CD137 agonist monoclonal antibodies (mAbs) show a potent anti-tumor activity (6). Paradoxically, however, stimulation of CD137 results in strong suppression of a variety of autoimmune or inflammatory diseases that are believed to be mediated mainly by CD4^+^ T cells (7-19). Although there is no consensus on how stimulation of CD137 inhibits disease, most evidence suggests that its effects are linked to decreased activity of pathogenic CD4^+^ T cells. Therefore, signaling through CD137 seems to be differentially regulated in CD4^+^ T cells and CD8^+^ T cells.

There is accumulating evidence showing that CD137/CD137L interactions are involved in inflammation (20,21). These interactions may regulate inflammation by forming a complex network connecting various immune cells and non-immune cells. In this brief Review, I focus on the role of CD137/CD137L interactions in inflammation involving non-T cells, including granulocytes, macrophage and endothelial cells. I also discuss how signaling through CD137 and CD137L result in inflammation, for which interference of the CD137 and CD137L pathway may provide a promising therapeutic tool. Specific emphasis is put on the reverse signaling through CD137L that is thought to be linked to inflammation, even though this area is not broadly studied.

## EXPRESSION OF CD137 AND CD137L

High levels of CD137 are constitutively expressed on FoxP3^+^ regulatory CD4^+^ T cells (22,23). Expression levels of CD137 on other types of cells are none, very low, or upregulated under a certain condition such as inflammation. Except for FoxP3^+^ regulatory CD4^+^ T cells, CD137 expression is inducible in lymphoid cells, including CD4^+^ and CD8^+^ T cells (1-3), natural killer (NK) cells (24), and NKT cells (25). Among myeloid cells, neutrophils seem to constitutively express CD137, even though its levels are low on the cell surface (26,27). CD137 expression also is induced on other granulocytes such as mast cells (28) and eosinophils (29), and probably on inflammatory macrophages (30). Interestingly, CD137 is inducible on activated myeloid progenitors (31). Recent studies have identified expression of CD137 on nonhematopoietic cells under disease conditions. These include endothelial cells, smooth muscle cells, and cardiac myocytes (20,32-34).

CD137L is constitutively expressed on professional antigen-presenting cells (dendritic cells, monocyte/macrophages, and B cells) and upregulated by their respective activation stimuli (1-3). Like CD137, CD137L can be expressed in cardiovascular components such as endothelial cells, smooth muscle cells, and cardiomyocytes (20,30,32,34,35). CD137L is detectable on hematopoietic stem cells (31,36,37). Our data have shown that CD137L expression is observed on various cell types, including neutrophils and fibroblasts (our unpublished data). Expression profile of CD137/CD137L is summarized in Table I.

## AMPLIFYING INFLAMMATORY RESPONSES THROUGH NON-T CELLS

There is evidence supporting that CD137/CD137L interactions participate in inflammation regulated by CD4^+^ T cells. For example, various inflammatory/autoimmune diseases (rheumatoid arthritis, autoimmune myocarditis, herpetic stromal keratitis, allograft rejection, and graft-versus-host disease) have a milder disease severity in the absence of CD137/CD137L interactions (11,21,30,38-42). However, it is not clear that the decreased inflammation is directly attributable to blockade of the CD137 costimulatory signal transduction pathway in CD4^+^ T cells. Instead, CD137/CD137L interactions may control other steps of inflammatory processes rather than the activation phase of CD4^+^ T cells. There is evidence supporting this explanation. First, CD4^+^ T cells of CD137-deficient mice show a hyperresponsiveness to antigens (higher proliferation and effector function) after antigen exposure (43). Second, CD137/CD137L interactions play a role in inflammation mediated by NK cells or NKT cells (25,44). Third, many studies suggest that signaling via CD137 or CD137L is important for the differentiation and function of myeloid cells, including granulocytes, a major type of inflammation players (27-29,31,33,35).

CD137-deficient neutrophils are defective in phagocytosis and ROS production, which are associated with the susceptibility of CD137-deficient mice to *Listeria monocytogenes* infections (31). The defects of CD137-deficient neutrophils might be due to their inability to deliver activation signals. Our data also show that CD137-deficient neutrophis is not able to infiltrate into the kidney after ischemia-reperfusion injury (our unpublished data). Interestingly, despite their normal trafficking ability to the kidney that receives ischemia-reperfusion injury, CD137-sufficient neutrophils were not able to damage the kidney of CD137-deficient mice. These results indicate that signaling through CD137L on neutrophils is required for tissue damage mediated by neutrophils. It is possible that the cytoplasmic domain of CD137L directly interacts with docking proteins in granules of neutrophils, resulting in degranulation, since proteins present on vesicles are shown to interact with the cytoplasmic domain of CD137L (HW Lee, personal communication). CD137-deficient mast cells also show defects in signaling and degranulation triggered by IgE (28). In contrast with these pro-inflammatory properties of CD137, CD137 on human neutrophils exhibits anti-inflammatory activity by abrogating GM-CSF-mediated anti-apoptosis (27). The reason why this difference occurs between human beings and mice is not known. In a similar context with neutrophils, activation of CD137 blocks GM-CSF or IL-5-mediated anti-apoptosis of eosinophils in patients with atopic dermatitis and extrinsic asthma (29). In contrast, CD137 is not expressed on eosinophils of patients with intrinsic asthma and idiopathic eosinophilia, therefore implicating that the absence of CD137 signaling is associated with the accumulation of eosinophils.

Non-hematopoietic cells of the host are required for induction of acute lethality by anti-CD137 mAb in graft-versus-host disease (42), which indicates that signaling through CD137 in non-hematopoietic cells play a critical role in inflammation induced by radiation and donor T cells. Indeed, CD137 expression is inducible by proinflammtory cytokines in endothelial cells, and activation of CD137 induces adhesion molecule expression on their cell surface (20,21). Reverse signaling through CD137L enhances monocyte migratory activity (20). Therefore, it is possible that signaling through either CD137 or CD137L guides inflammatory cells to the inflammatory site.

Considering that CD137/CD137L interactions are critical in the induction of inflammation, blocking of CD137/CD137L should inhibit the progression of inflammatory diseases. In fact, this is the case (30,32,38,39,44). However, it is not known whether the blocking effects are due to the absence of signaling through CD137 or CD137L. As discussed in the next section, CD137L reverse signals might turn out to be mainly responsible for mediating inflammation.

## REVERSE SIGNALING, INFLAMMATION, AND DIFFERENTIATION OF MYELOID CELLS

It is now being accepted that many members of the TNF superfamily, including CD137L, have a reverse signal transduction pathway (45). Accumulating evidence indicates that CD137L signals regulate the activity of professional antigen-presenting cells. For example, a recent study has demonstrated that engagement of CD137L induces B cell proliferation and immunoglobulin production (46). In particular, there are prominent phenotypic changes in monocytes that receive CD137L stimulation, including cell proliferation, production of proinflammatory cytokines and chemokines, and upregulation of cell adhesion molecules (thus enhancement of their infiltration capacity) (reviewed in Ref 47). Signaling via CD137L mediates similar activities in dendritic cells that are observable in monocytes: Cross-linking of CD137L enhances the expression of costimulatory ligands and MHC molecules and the release of cytokines (47,48). In sum, CD137L signals on antigen-presenting cells act towards enforcing the adaptive immune response and inflammation.

Hematopoietic progenitor cells of CD137- or CD137L-deficient mice have greater differentiation potency for myelopoiesis (31). This is due to the absence of CD137L signaling. In a similar context, CD137 on osteoblasts inhibits M-CSF/RANKL-mediated osteoclast differentiation by engaging CD137L on their cell surface (49). Some studies reported contradictory results by showing that engagement of CD137L induces proliferation of hematopoietic progenitor cells and differentiation to macrophages (36,37). The physiological meaning underlying these observations is not known yet.

Evidence barely exists that signaling through CD137L regulates inflammation in vivo. As mentioned previously, our reconstitution experiment indicates that effector mechanisms of neutrophils involve the CD137L but not CD137) signal transduction pathway in ischemia-reperfusion kidney injury (our unpublished data). Furthermore, we have demonstrated that specific blocking of the CD137L signaling pathway, using TAT-CD137L cytoplasmic domain fusion protein (which blocks the signal transduction delivered by endogenous CD137L), induces increased vascular permeability in vivo, a phenomenon which is observed in CD137-deficient mice and in mice that receive anti-CD137L mAb (our unpublished data). Therefore, it is now clear that signaling through CD137L is critical in regulating inflammation in various ways.

Molecules involved in the CD137L signal transduction pathway are now being indentified. Upregualtion of cytokines and cell adhesion molecules in monocytes after CD137L stimulation specifically requires AKT and mTOR/p70S6 kinase (50). Preliminary results show that the cytoplasmic domain of CD137L bind to proteins potentially associated with degranulation and phosphorylation, indicating that the cytoplasmic domain of CD137L may function as a docking partner of granule membrane proteins to trigger degranulation, as well as form a complex with protein kinase to deliver downstream signals leading to production of cytokines in granulocytes and monocytes (HW Lee, personal communication). A recent study has shown that CD137L forms a complex with TLRs and sustains TNF production by TLRs, independently of engagement with CD137 (51).

## CONCLUSION REMARKS

**Figure 1:**
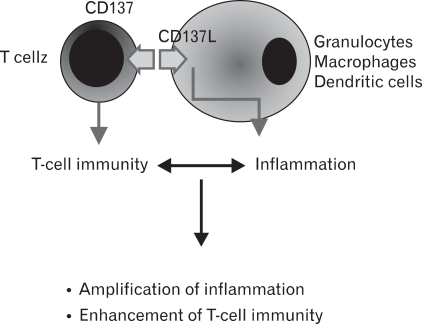
A schematic diagram showing a bidirectional signal transduction of the CD137 and CD137L pathway.

Humanized anti-CD137 mAb is in Phase II clinical trials to treat melanoma patients, and its use is being evaluated in treating autoimmune and other inflammatory diseases and as adjuvants for vaccines. A major obstacle to more effective use of the CD137 pathway in clinical use is that we still do not completely understand the mechanisms of CD137-mediated immune suppression and immune stimulation. In addition, bidirectional signaling complicates the biology of the CD137 receptor/ligand system. It is urgent that we should define which activities are mediated by CD137 and which by CD137L. Development of tools to specifically block CD137 or CD137L signals will be of great help in clarifying this issue. A particularly challenging question is how CD137/CD137L interactions simultaneously enhance inflammation and T-cell immunity. I suggest that signals delivered through CD137L or CD137L protein itself may play a critical role in amplifying inflammation such a way that CD137L signals induce the production of proinflammatory cytokines and chemokines, probably in synergy with TLR signals and by triggering degranulation of granulocytes and other myeloid cells (Fig. 1). Inflammation amplified by CD137L in turn may contribute to enhancement of T cell activities together with CD137 signals by increasing expression levels of other costimulatory molecules, MHC, cell adhesion molecules, and cytokines in antigen-presenting cells. Thus, CD137/CD137 interactions form an mutually amplifying loop of inflammation and T-cell immunity. It is expected that future studies will form a new paradigm for the biology of CD137/CD137L.

## Figures and Tables

**Table I:**
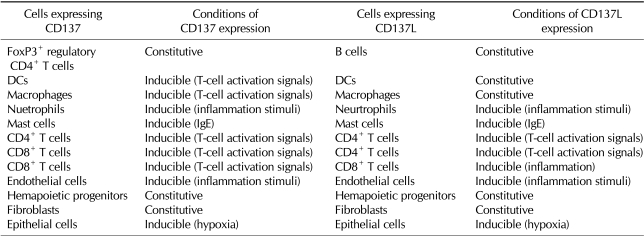
Expression profile of CD137 and CD137L
